# The Global Interest in Vaccines and Its Prediction and Perspectives in the Era of COVID-19. Real-Time Surveillance Using Google Trends

**DOI:** 10.3390/ijerph18157841

**Published:** 2021-07-24

**Authors:** Magdalena Sycinska-Dziarnowska, Iwona Paradowska-Stankiewicz, Krzysztof Woźniak

**Affiliations:** 1 Department of Orthodontics, Pomeranian Medical University in Szczecin, Powstańców Wielkopolskich Street 72, 70111 Szczecin, Poland; krzysztof.wozniak@pum.edu.pl; 2 Department of Epidemiology of Infectious Diseases and Surveillance, National Institute of Public Health—National Institute of Hygiene, 00791 Warsaw, Poland; istankiewicz@pzh.gov.pl

**Keywords:** vaccination programs, COVID-19 vaccine, flu vaccine, BCG vaccine, HPV vaccine, pneumococcal vaccine, Polio vaccine, Google Trends

## Abstract

Background: The COVID-19 pandemic has globally overwhelmed all sectors of life. The fast development of vaccines against COVID-19 has had a significant impact on the course of the pandemic. Methods: Global data from Google Trends was analyzed for vaccines against flu, BCG, HPV, pneumococcal disease, polio, and COVID-19. The time frame includes the last five-year period starting from 17 April 2016. Multiple training of time series models with back testing, including Holt–Winters forecasting, Exponential Smoothing State Space, Linear model with trend and seasonal components (tlsm), and ARIMA was conducted. Forecasting according to the best fitting model was performed. Results: Correlation analysis did not reveal a decrease in interest in vaccines during the analyzed period. The prediction models provided a short-term forecast of the dynamics of interest for flu, HPV, pneumococcal and polio vaccines with 5–10% growth in interest for the first quarter of 2022 when compared to the same quarter of 2021. Conclusions: Despite the huge interest in the COVID-19 vaccine, there has not been a detectable decline in the overall interest in the five analyzed vaccines.

## 1. Introduction

In March 2020, the WHO announced the COVID-19 pandemic [1], the unprecedented situation had an impact on almost all sectors of life. The COVID-19 disease may remain asymptomatic or may develop symptoms mainly of the upper respiratory tract, and people who have recovered are burdened with numerous long-term complications of the respiratory, cardio-vascular and nervous systems. On the other hand, some patients progressed into severe pneumonia with ARDS, which may cause death; the disease was noted to be particularly dangerous for older persons [2]. At the end of May 2021, it was reported that more than 3.5 million people had died due to the COVID-19 virus, and more than 168 million cases of the disease were noted all over the world [3].

After the outbreak of the COVID-19 pandemic, the search for an effective vaccine began. Since only an effective vaccine would be able to slow or stop the spread of the virus, and because it was believed it would take more than a year to develop a new vaccine, other measures were taken to prevent the spread of the virus. Vaccines against COVID-19 were first distributed worldwide at the beginning of 2021, and this process of vaccination has begun in earnest globally [4,5].

According to the study conducted by Paguio et al. [6], changes in health behaviors during pandemics, coupled with the reaction to COVID-19, can make vaccines for other diseases a lesser priority and make it difficult to assess the impact of vaccination programs. Before the COVID-19 vaccines were released, the effectiveness of existing vaccines against the new virus was actively explored, with the BCG vaccine receiving special consideration [7]. The inquiry in question was whether or not the BCG vaccination could protect against COVID-19 or at least reduce the severity of the disease. It was observed during the pandemic that the BCG vaccine was suspected to have some impact or could evoke cross-immunity in regards to COVID-19 [8,9,10]. A similar pattern was observed in that after the flu vaccination, patients had significantly less COVID-19 morbidity than the unvaccinated group. The hazard ratio was 0.74 (95% CI: 0.54–0.89) [11]. On the other hand, there was a threat of recurrence of other diseases, such as polio, because there could be a reduction in vaccinations due to the pandemic and due to the focusing of resources on a vaccine against COVID-19 [12].

In the research, multiple types of vaccines for respiratory diseases and against illnesses not related to the respiratory system, such as HPV, were analyzed to show interest in different types of vaccines during the COVID-19 pandemic and to predict interest in the analyzed vaccines. If, due to the COVID-19 pandemic, there is a change in vaccination interest, public health institutions may become better prepared for that situation. Campaigns to encourage vaccination could be provided in cases of lack of interest in other vaccines during the COVID-19 pandemic. On the other hand, if there is greater interest in vaccination programs, health institutions should prepare and take advantage of this fact when considering their vaccination strategy.

Google Trends (GT) is known as the most popular and useful tool for exploring online human behavior, as well as predicting disease outbreaks or occurrence [13]. The use of GT in scientific articles is still novel; however, it is highly accepted and discussed by scientists, with papers published in different fields [14,15,16,17,18,19,20].

In view of the insufficient research on the subject of global interest in other vaccines in the era of COVID-19, the aim of this study was to find out and predict if the increased interest in the COVID-19 vaccine caused a decrease of interest in other vaccines.

## 2. Materials and Methods

The Relative Search Volumes (RSV) from GT [21,22] of Internet search interest among anonymous Google search engine users was analyzed with consideration of the time of the vaccination research, including the last 5 years starting from 17 April 2016. The data used for the analysis were already anonymized, categorized and aggregated by Google algorithms. The dynamics of changes in the number of requests on the whole set was analyzed. The data for the whole world were analyzed for vaccines against flu, BCG, HPV, pneumococcal disease, polio, and COVID-19. Data samples for statistical analysis was collected from GT for individual phrases such as: “flu vaccine”, “BCG vaccine”, “HPV vaccine”, “pneumococcal vaccine”, “Polio vaccine”, “COVID-19 vaccine”, queried by users in the Google search engine. In total, there were six data collections downloaded and given shorter names for simplicity: “flu”, “bcg”, hpv”, “pneumo”, “polio” and “covid” respectively. One data observation represents a relative number of queries within a single week with a range of values from 0 to 100, where 100 stands for the maximum number of searches in a collected period of time. With near seven billion queries stated per day in the Google search engine, the sample from GT for the analyzed period of time concerns a large sample size which is adequate for scientific study [23]. Brief descriptive statistics by collection are shown in Table 1.

All statistical analyses with data visualization have been programmed in the interpreted programming language “R” [24] with the open-source software for data science, scientific research, and technical communication “R studio” version 1.4.1106 (RStudio, PBC: Boston, MA, USA, 2020) [25].

In regards to the aim of the study to find out if the increase of interest in COVID-19 vaccines could cause decreased interest in other vaccines, correlation analysis [26] was applied to identify and quantify the relationship between the two collections. Thus, the correlation parameters have been measured by alternate pairwise comparisons of the “covid” collection and a collection from the following set: “flu”, “bcg”, “hpv”, “pneumo”, “polio”. The correlation has been analyzed based on the following aspects:

1. Visualization of the time series data [27,28],
2. Lags analysis by autocorrelation function (ACF) and partial autocorrelation function (PACF) [29],
3. Causality by cross-correlation function.

The visualization was done pairwise, where time series plots were presented for a visual description of cycles, trends and seasonality. The analyses of the correlation between a series and its lags were done because some of the past lags may contain predictive information and such information can be utilized to forecast events of the series. To quantify those relationships, the level of correlation between the series and its different seasonal lags was measured by ACF and PACF. The goal of the causality analysis, in the context of the time series analysis, was to identify whether a causal relationship exists between the series we wish to forecast due to other potential exogenous factors. The correlation type and strength between the time series was conducted using a cross-correlation function (CCF) plot. 

Several models for data forecasting, such as Autoregressive Integrated Moving Average (ARIMA), make a general assumption of stationarity of the time series; therefore, stationarity of data was checked. Weak stationarity is also an assumption for performing ACF. For this reason the Augmented Dickey–Fuller (ADF) [30] t-statistic test was performed to find if the series has a unit root, with the following null hypothesis test: if p-value is less than 0.05, the null hypothesis can be rejected and it can be assumed that the data is stationary and an alternative hypothesis test applied. If the *p*-value is more than 0.05, then we fail to reject the null hypothesis and determine the data to be non-stationary. 

The forecasting process algorithm was executed in the following steps [31,32]:

1. creation of testing and training partitions,
2. visualization of forecast performance,
3. training multiple time series models with back testing, including Holt–Winters forecasting, Exponential Smoothing State Space, Linear model with trend and seasonal components (tlsm), and ARIMA,
4. visualizing the models’ performance,
5. forecasting according to the model with the best performance.

A 12-month prediction was performed for the vaccine queries where it was applicable.

## 3. Results

**Figure 1:**
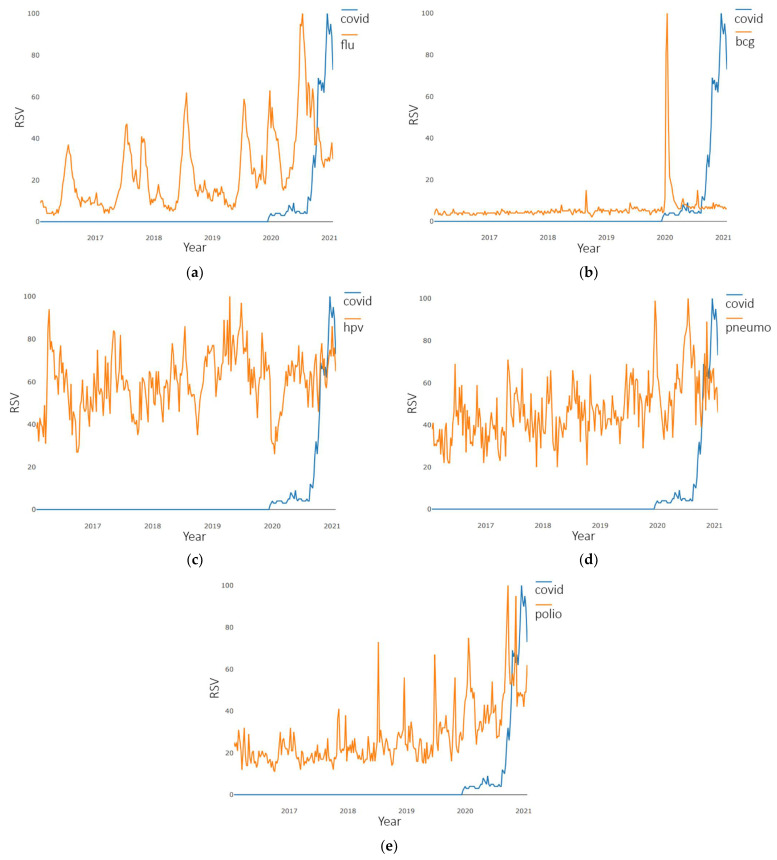
Pairwise time series plots of weekly number of requests (2016–2021) for: (**a**) “covid” and “flu”; (**b**) “covid” and “bcg”; (**c**) “covid” and “hpv”; (**d**) “covid” and “pneumo”; (**e**) “covid” and “polio”.

Pairwise time series plots of “flu”, “bcg”, “hpv”, “pneumo”, and “polio” collections along with the “covid” collection are presented in Figure 1.

In Figure 1a an increasing trend for both “flu” and “covid” data samples can be noticed; with the “flu” time series affected by seasonal factors (with yearly peaks between October and December), cycles were not detected. Figure 1b shows a pronounced trend only for the “covid” sample, while the “bcg” trend stays practically unchanged with no seasonality with only a single major cycle at the end of March 2020. In Figure 1c, a pronounced trend can be seen only for the “covid” sample, while the “hpv” trend remains unchanged with seasonality in the middle of the year with no visually noticeable cycles. One can notice a moderately growing trend for “pneumo” in Figure 1d, which also represents data with no seasonality but with few pronounced cycles since the end of 2019. In Figure 1e there is an observable pronounced trend for the “covid” sample, while the trend for “polio” data changed from almost unchanged (to the first half of 2018) to growing almost exponentially with seasonality (end of the year) and a few pronounced cycles starting from the second half of 2018.

**Figure 2:**
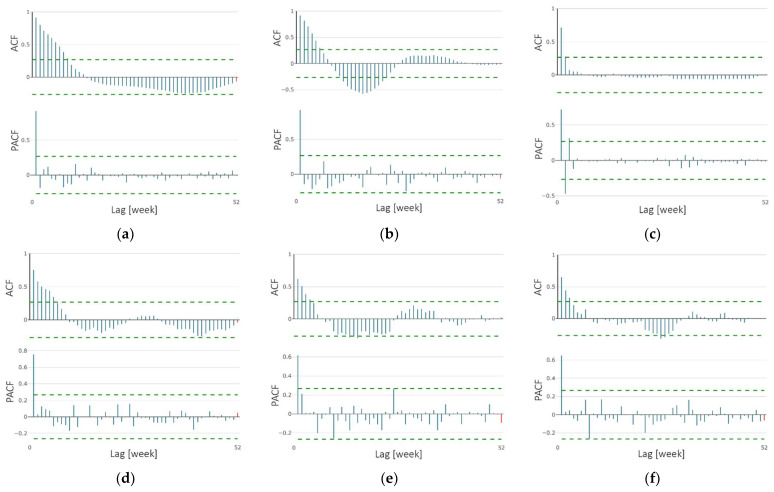
ACF and PACF plots of time series for (**a**) “covid”; (**b**) “flu”; (**c**) “bcg”; (**d**) “hpv”; (**e**) “pneumo”; (**f**) “polio”.

The ACF and PACF plots are shown in Figure 2. It can be seen from the ACF plots that the series are highly correlated with the close lags. A large spike can also be seen at lag 1 that decreases after a few lags. A significant correlation can be seen on the PACF plots at the first lag (for “bcg” time series also at second and third), followed by correlations that are not significant, which represent an autoregressive term of order 1 (for “bcg” of order 3) in the data. Based on the obtained correlation results, the data for all the time series are not independent; therefore, an ARIMA model for predictions could be applied.

**Figure 3:**
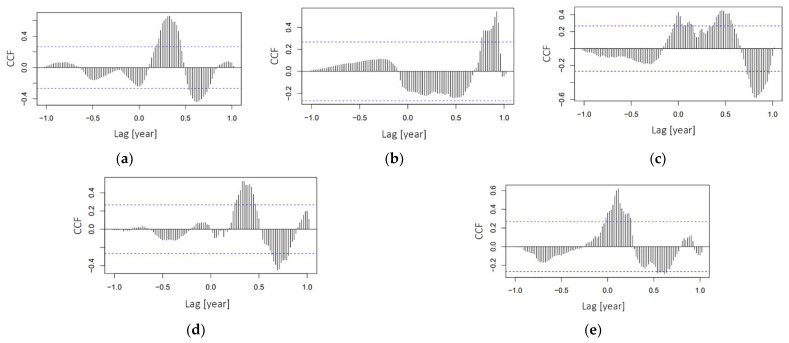
Cross-correlation plots for: (**a**) “covid”–“flu”; (**b**) “covid”–“bcg”; (**c**) “covid”–“hpv”; (**d**) “covid”–“pneumo”; (**e**) “covid”–“polio”.

The cross-correlation plots are presented in Figure 3. It can be seen that the cross-correlation of “covid”–“flu” is changeable over time, from maximum negative scores (up to 0.45 on lags interval (0.5–0.8)) to maximum positive scores (up to 0.55 on lags interval (0.15–0.5)). Currently, the growth of “covid” requests negatively correlates (negligible but close to significant level) with “flu” requests. It is also discernible that the “covid”–“bcg” cross-correlation (as shown on Figure 3b) is also changeable over time: the maximum positive values (around 0.5) appeared on interval (0.7–1), this is due to a very pronounced “bcg” cycle at the beginning of 2020, after which the cross-correlation is permanently negative (but also non-significant). This negative correlation is explained by the persistence of the “bcg” trend from 2020 without further bursting cycles and the increasing trend of “covid” requests. It can be seen that the “covid”–“hpv” cross-correlation (see Figure 3c) is also changeable over time: the maximum negative values (around −0.6) were on interval (0.7–1), after which the cross-correlation has only positive values (predominantly significant) with maximum values near 0.4. The dynamics of interest in “covid” and “hpv” vaccines changes unidirectionally. In the “covid”–“pneumo” cross-correlation plot shown in Figure 3d it can be seen that the cross-correlation changed many times during the period under review. Since 2021, any changes of interest in “covid” vaccine do not affect changes of interest in “pneumo”. In the case of “covid”–“polio” cross-correlation, a pronounced variability of the correlation is also noticeable with increasing amplitude from moderately negative in the first half of 2020 to strong positive from the second half of 2020 to first quarter of 2021.

The “covid” data could not be predicted due to the too short observation period—less than 2 years. Results of the ADF test for each collection are grouped in Table 2.

The *p*-value is less than 0.05 for all queries, thus, the null hypothesis for all data collections was rejected and the data is stationary; therefore, ARIMA models for data forecasting could be applied without additional transformation.

The forecast of “bcg” data was not performed due to data fluctuations in the analyzed period. The list of best training models for each considered vaccine is shown in Table 3.

**Figure 4:**
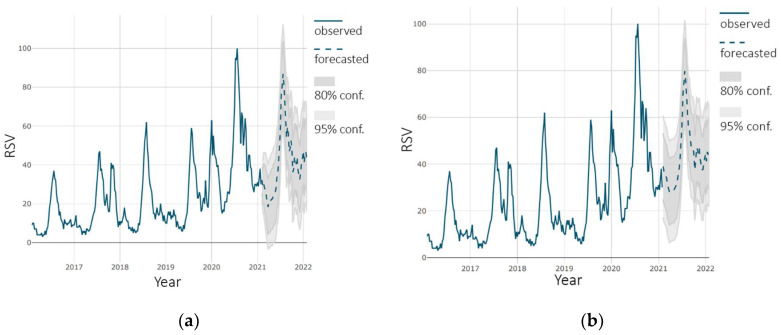
Forecast of interest in “flu” vaccine: (**a**) using SARIMA model; (**b**) using tslm model.

A prediction is presented as a dashed line on dark gray and light gray background (80% and 95% confidence respectively). The prediction for “flu” vaccine is shown in Figure 4. The forecast shows an increase in the level of interest in vaccine. The growth rate at the end of the forecast period ranges from 5% to 7% compared to the same period in 2021. Both models used gave very similar results.

**Figure 5:**
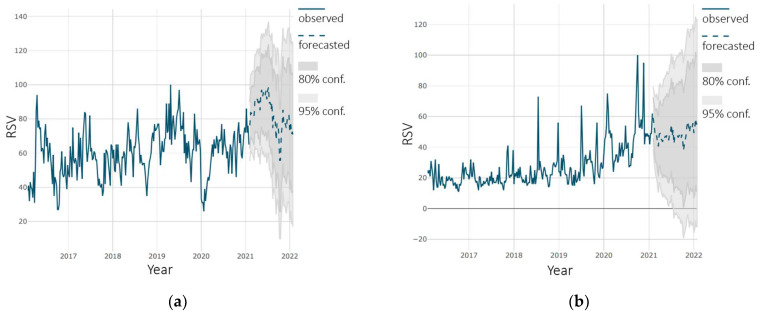
Forecast using Holt–Winters model of interest in: (**a**) “hpv” vaccine; (**b**) “polio” vaccine.

The prediction for the HPV vaccine is shown in Figure 5a. According to the figure, the level of interest in the vaccine at the end of the forecast period is at the same level as at the beginning of 2021 (the difference is within the boundaries of statistical error). 

The prediction for the polio vaccine is shown in Figure 5b. According to the figure, the forecast shows an increase in the level of interest in vaccine. The growth rate at the end of the forecast period is in the range from 7% to 10% compared to the same period in 2021.

**Figure 6:**
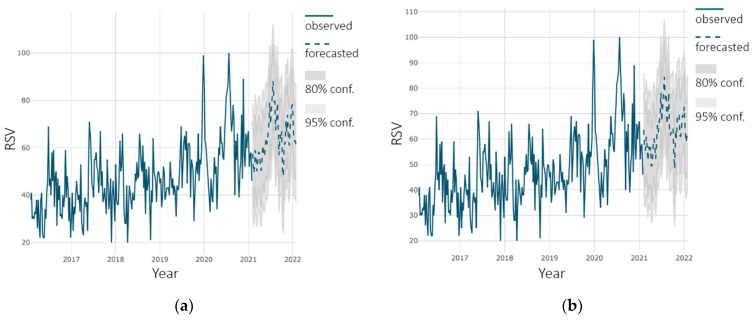
Forecast of interest in “pneumo” vaccine: (**a**) by using SARIMA model; (**b**) using tslm model.

The prediction for the pneumococcal vaccine is shown in Figure 6. According to the figure the forecast shows an increase in the level of interest in the vaccine. The growth rate at the end of the forecast period is in the range of 5% to 10% compared to the same period in 2021. Both models give almost the same results.

The correlation analysis did not reveal a decrease in interest in vaccines during the analyzed period. The prediction models provided a short-term forecast of the dynamics of interest for “flu”, “hpv”, “pneumo” and “polio” vaccines which showed a 5% to 7% and 5% to 10% growth in interest for the first quarter of 2022 compared to the same quarter of 2021, depending on the vaccine.

## 4. Discussion

The aim of the study was to find out and predict if the increased interest in the COVID-19 vaccine could cause a decrease of interest in other vaccines. To the best of our knowledge, this is the first study to discuss the impact of COVID-19 vaccination on interest in other vaccines in the Google search engine. The first noticeable value of the research is that, with the great interest the COVID-19 vaccine, interest in other analyzed vaccines had not declined. 

Previous literature suggested the greater interest in the BCG vaccination [3,4], which is in line with the results of our study; a more pronounced interest in the BCG vaccine was detected, with a peak regarding the BCG vaccine queries occurring in April 2020. However, overall interest in the BCG vaccine has remained unchanged. According to Fu et al. [7], the BCG may offer some protection against COVID-19; however, it may differ over time, depend on the age structure, and vary in different populations. The viral pathogens often cause respiratory tract infections in children. Stensballe et al. [33] proved that BCG reduced the occurrence of respiratory syncytial virus infection. A similar preventive effect of the BCG vaccine was found among older individuals in Indonesia [34]. In the most recent study, a 70% decrease in respiratory tract infections due to the BCG vaccination was reported [35]. The induction of trained immunity by the BCG vaccine may protect against COVID-19, but, according to the authors, this hypothesis needs more testing in randomized clinical trials [8]. 

Vaccination programs in Pakistan and Afghanistan, countries where the polio virus is still endemic, were suspended in April 2020 due to the COVID-19 pandemic, which lead to forty million children left without the polio vaccination [12]. Apart from the 99.9% decrease in polio cases since 1988, polio still remains a public health problem. Barriers to polio vaccination connected with the COVID-19 pandemic caused a growth in polio cases, with 1226 cases reported in 2020, compared to 138 cases of all forms of polio recorded in 2018. The WHO strategy underscores efforts to offer a comprehensive set of actions, such as routine immunization, as well as forming closer partnerships with communities at high risk of polio, in order to even better meet their health needs, particularly in Afghanistan and Pakistan [36]. Quite unexpectedly, our study found greater interest in the polio vaccination, the threat of the polio vaccination being suspended globally in the era of COVID-19 vaccination was not confirmed in the performed analysis. 

The risk of the COVID-19 disease may be reduced by immunization with the influenza vaccine [11]. During global COVID-19 vaccination programs, the appeal for vaccinations against other respiratory diseases that have some similar symptoms to COVID-19 may occur [37]. The global threat of COVID-19 may lead to greater interest in vaccines for other diseases [6]. This is in line with the conducted study; during the pandemic, interest in other vaccines may increase with regard to illnesses with symptoms similar to the pandemic. As flu interest seasonality was observed in previous studies of [37,38], it was also detected in our study. The World Health Organization recommends the use of seasonal flu vaccines, especially for adults over 65 years and pregnant women, as well as people with chronic illnesses [4,5,39]. Furthermore, through indirect protection, vaccines against seasonal influenza and pneumococcal infections globally, which are included in almost all routine immunization programs, have partially eliminated the pathogens as a hazard to the older population [40]. According to previous studies, the pneumococcal vaccination implemented among older persons could prevent up to 33–40% of the pneumococcal disease and potentially pneumococcal morbidity and mortality. Both seasonal pneumococcal and the influenza vaccine can prevent a substantial burden of mortality among adults at-risk. When the COVID-19 pandemic occurs simultaneously with the flu season and the pneumococcal disease, high-coverage vaccination of the population will bring benefits such as a decrease in the number of hospitalizations due to pneumococcal or influenza diseases and can reduce the frequency of urgent treatment needed for non-COVID-19 patients [37]. Moreover, the similarity of symptoms among the upper respiratory track diseases can entail an increase of the potential COVID-19 survival rate, due to the lower risk of co-infections, among persons vaccinated for other upper respiratory diseases [4,5]. Similarly, through herd effects of maintaining vaccine coverage of existing vaccine programs for children, a reduction in the rate of the associated disease in the older population may occur, as well as an increase in benefits for limiting COVID-19 risks. In our study, this thesis may be supported by the rise in interest in queries regarding vaccinations. According to our predictions there will be greater interest in most of the investigated vaccines (excluding the BCG vaccine), showing 5–10% growth rates when compared to the first quarter of 2021. Our results are compatible with the study conducted by Paguio et al. [6], where the researchers proved that the reaction to COVID-19 pandemic can change interest in vaccines for other diseases. Moreover, in our research, no decline in the interest in the HPV vaccine was observed, which can be perceived as a positive finding due to the fact that interest in this vaccine represents concern regarding non-respiratory diseases.

There are some limitations associated with this analysis. First, the use of GT as the only data set does not contain all of the available Internet search data. On the other hand, the Google search engine constitutes 92% of search activity [41,42]. Secondly, due to the anonymity of the data, it is hard to determine which parts of the population may be excluded or underrepresented in the study [43]. It should be noted that the presented data and current analysis cannot be automatically generalized for the whole population. It reflects only a trend for users of Google services. It does not represent the interest of people without Internet access. To generalize the results obtained, additional research is required.

## 5. Conclusions

Firstly, according to the analysis of data from GT, along with the huge interest in the coronavirus vaccine, there has been no decline in overall interest in the five analyzed vaccines.

Moreover, the overall interest in “bcg” and “hpv” vaccines among Google search engine users, since the coronavirus era begun, has remained largely unchanged, while popularity of other vaccines has increased and varies from negligible (“flu” and “pneumo”) to significant (“polio”).

Finally, the predicted dynamics of interest in vaccines based on GT data did not confirm the decline in the popularity of vaccines. On the contrary, for three out of the four vaccines, their growth in popularity is predicted to grow at rates of 5–10% relative to the 1st quarter of 2021. 

## Figures and Tables

**Table 1:** Descriptive statistics of time series data collections.

Collection	Frequency [Weeks]	Start Time	End Time	Total Count [Weeks]
covid	52	8 March 2020	11 April 2021	58
flu, bcg, hpv, pneumo, polio	52	17 April 2016	11 April 2021	261

**Table 2:** Augmented Dickey–Fuller test results.

Collection	Dickey–Fuller	Lag Order	*p*-Value
flu	−4.09	6	<0.01
pneumo	−5.17	6	<0.01
hpv	−4.43	6	<0.01
bcg	−5.11	6	<0.01
polio	−4.13	6	<0.01

**Table 3:** List of training model according to the best performance.

Vaccine	Model
flu	SARIMA, tslm
hpv	Holt-Winters
pneumo	SARIMA, tslm
polio	Holt-Winters

## Data Availability

The data presented in this study are available on request from the corresponding author.
